# Income-related inequality in smoking cessation among adult patients with cardiovascular disease: a 5-year follow-up of an angiography intervention in Luxembourg

**DOI:** 10.1186/s12872-017-0541-2

**Published:** 2017-05-05

**Authors:** Anastase Tchicaya, Nathalie Lorentz, Stefaan Demarest

**Affiliations:** 1 Luxembourg Institute of Socio-Economic Research (LISER), Living Conditions Department/Health Research Team, Esch-sur-Alzette, Luxembourg; 2 Scientific Institute of Public Health WIV-ISP, DO Santé publique et surveillance, Brussels, Belgium

**Keywords:** Income inequality, Cardiovascular diseases, Smoking cessation, Luxembourg

## Abstract

**Background:**

Smoking contributes to cardiovascular diseases (CVD), a leading cause of death and a large source of healthcare costs in Western countries. We examined the association between income and smoking cessation among smokers who underwent coronary angiography at the National Institute for Cardiac Surgery and Interventional Cardiology in Luxembourg.

**Methods:**

Data were derived from a follow-up study conducted in 2013/2014 among 4391 patients (of which 1001 patients were smokers) at the time of coronary angiography in 2008/2009. Four logistic regression models were applied. In three models, the predictor was income and the covariates were sex, age, nationality, marital status, diagnosis, body mass, physical activity, and awareness of tobacco as a cardiovascular (CV) risk factor. In the other model, the predictor was an interaction term composed of income and awareness of tobacco as a CV risk factor; the other variables were covariates.

**Results:**

Among patients who were current smokers at baseline, 43.2% were current smokers at follow-up and 56.8% had quit smoking. In the multivariate logistic models, quitting smoking was associated with income even after controlling for socio-demographic, diagnostic, and behavioural risk factors. In the full model, the odds of quitting smoking among patients in the two highest income categories remained significant when compared to patients in the lowest income category: odds ratio (OR) = 2.8; 95% confidence interval (CI), 1.3–6.1 and OR = 2.8; 95% CI, 1.2–6.5, respectively. In the full model with an interaction term, quitting smoking was only associated with income when patients knew tobacco was a CV risk factor. The odds of smoking cessation were 5.62 (95% CI: 2.13–14.86) and 3.65 (95% CI: 1.51–8.86) times for patients with annual incomes of 36,000–53,999€ and ≥54,000€, respectively), compared to those for patients with an annual income of <36,000€.

**Conclusions:**

This study highlights the influence of income on behaviours regarding CVD risk factors after a major CVD event. Patients in the highest income groups were more likely to quit smoking, although only when they were aware of tobacco as a CV risk factor. Therefore, intervention strategies targeting lower income groups should be implemented in major health facilities.

## Background

Smoking contributes extensively to cardiovascular diseases (CVD), which are the leading causes of death and the second largest source of healthcare costs in Western countries [1, 2]. Continuing to smoke after a coronary event is associated with mortality levels that are significantly higher than the mortality levels among those who quit. In addition, smoking cessation rapidly reduces the risk of a coronary event once smoking stops, with the risk of such an event approaching that of non-smokers by 3 years after cessation [3–5]. A meta-analysis of cohort studies assessing the effects of smoking cessation on mortality after a myocardial infarction estimated that the combined odds ratio of death for smoking cessation compared with smoking continuation was 0.54 (95% confidence interval [CI], 0.46–0.62) [6]. Furthermore, quitting smoking has been associated with a substantial reduction (36%) in the risk of all-cause mortality among patients with CVD [7].

Nevertheless, there is evidence that a substantial number of patients who were smokers prior to hospitalisation for coronary heart disease continue to smoke after discharge, despite advice to stop [7–9]. Although it could be presumed that hospitalisation serves as an important event—a “teachable moment”—that could motivate smokers to quit smoking [10], this effect seems to be short term in many cases: most smokers resume smoking after hospitalisation [11].

Many factors are associated with the continuation of smoking among cardiac patients: younger patient age, higher level of nicotine dependency, lack of future intentions to quit [1], lack of will or confidence, absence of social support [12], belief that “the damage is done,” history of smoking relapse, hostility [13], and absence of smoking cessation advice [10, 14]. Furthermore, Berndt et al. [1] distinguished three types of hospitalised cardiac patients according to their smoking characteristics and predictors of smoking abstinence 1 month after discharge. The first and largest cluster (38.4% of patients) comprises patients with high scores on self-efficacy toward non-smoking, high scores on perceived social influence toward non-smoking, and the highest likelihood of successfully abstaining from smoking. The second and smallest cluster (26.4%) differs from the first one because its members reported the presence of many smokers in their social environments, but they showed high levels of smoking cessation. The third cluster—the hard-core smokers (comprising 35.2% of all patients)—had the highest risk of continuing or resuming smoking after hospital discharge and were characterised as smokers with low future intentions to quit [1].

Although the socio-demographic and psychological factors associated with smoking cessation or relapse have been studied in detail, the socio-economic factors that might affect smoking outcomes (e.g., educational level, income, occupational status) have been under evaluated. However, such factors have been shown to independently affect these outcomes [15]. Therefore, our objective was to examine the influence of income differences on smoking cessation success among patients who were smokers when they were admitted for a cardiac event at the National Institute for Cardiac Surgery and Interventional Cardiology (INCCI) in Luxembourg.

## Methods

### Data

Data were derived from a follow-up study of 4391 patients who underwent a coronary angiography at the INCCI in 2008/2009 and participated in the research project “Social Determinants and Health Status” [16]. Using the “Monitoring and Dynamics of health status through the Risk Factors for Cardiovascular diseases” project framework, patients were re-contacted by letter in June 2013 and asked to complete and return a self-questionnaire addressing physiological and behavioural CVD risk factors as well as socioeconomic and demographic characteristics [17]. In total, information on 1289 patients was available for use in the follow-up study, whereas at least 547 patients had died during the follow-up period (based on information provided by relatives and/or indicated on the questionnaire). One-to-one data linkage between the initial study and the follow-up study was possible because we used a unique identifier for every patient/participant.

A total of 1001 smokers were part of the initial 2008/2009 group, whereas 250 respondents in the 2013/2014 follow-up study were smokers. Despite the small number of smokers, the characteristics of the respondents who were smokers did not differ significant from the characteristics of non-respondents who were smokers.

The questionnaire used in the follow-up study was similar to the one used in the 2008/2009 study and addressed risk factors for CVD, such as overweight/obesity, lack of physical activity, smoking, high cholesterol levels, hypertension, and diabetes in addition to demographic and socio-economic data (age, gender, nationality, marital status, educational level, and occupation). Added to the questionnaire were items regarding household income and changes in lifestyle during the follow-up period.

### Indicators

#### Smoking cessation

Patients were considered to have successfully quit smoking (ex-smokers) if they indicated regular or occasional smoking on the initial 2008/2009 survey and not smoking on the follow-up survey, regardless of when they had quit smoking (i.e. recently or early in the follow-up period). Patients were categorised as current smokers if they indicated regular or occasional smoking on both the initial survey and the follow-up survey. Changes between regular and occasional smoking were not addressed.

#### Income

Household income level was used as a proxy for the socio-economic status of the patients. Data on the income level of the patients could only be derived from the follow-up survey, where patients could indicate the income category (8 categories) of their household. However, given the relatively high level of missing data for the income indicator (missing data for 180 patients), an imputation process was applied where, based on data for sex, age-group, and professional status, income level was imputed in cases of missing data. For each combination of sex, age-group, and professional status, the mean income based on the patients for which the income was known was imputed.

Patients were then categorised into three income groups: those with an annual household income of less than 36,000 euros (€), those with an income between 36,000 euros and 53,999 euros, and those with an income higher than 54,000 euros. The income cut-off points were chosen based on pragmatic reasons, assuring that each group covered (+/−) one-third of all patients.

#### Age

Because the majority of CVD patients in this study were older, patient age was divided into four categories: 54 or younger, 55–64 years, 65–75 years, and 75 years or older.

#### Nationality

Patients were classified as those with the Luxembourger nationality and those with another nationality.

#### Marital status

Married patients/patients in a partnership were distinguished from single patients, widowed, and divorced patients.

#### Diagnostic risk factors

Information on the diagnostic risk factors was derived from the initial 2008/2009 study. During the follow-up study, no diagnostic information was collected.

#### Cardiovascular risk factors

Information on cardiovascular (CV) risk factors was derived from the follow-up study and addressed only the status at follow-up. Changes in risk factors during the follow-up period were not assessed. Patients with a body mass index (BMI, based on self-assessed weight and height) ≥25 kg/m^2^ and <30 kg/m^2^ were classified as overweight, and those with a BMI ≥30 kg/m^2^ were considered obese. Physical activity was categorised in four groups: regular practice (30 min of physical exercise at least 3 times per week), occasional practice (30 min, less than 3 times per week), no physical activity due to health problems, and no physical activity for other reasons. Patients were asked if they were aware of the risk of using tobacco on their health. Based on this, two categories were distinguished: patients aware of the risk and patients not to be aware of the risk.

### Statistical analysis

In the first step, the composition of the sample at the initial study was compared with the composition of the sample at the follow-up regarding smoking status, household income, demographic data, diagnostic risk factors, and cardiovascular risk factors. Then, using a chi-square test (threshold *p*-value <10%), former smokers were compared with current smokers. The distribution of the awareness of tobacco as CV risk factor was analysed by smoking status.

To assess the association between smoking cessation and income, three consecutive logistic regression models without an interaction term were fitted, as well as one logistic regression model with an interaction term. In model 1, sex, age, nationality, marital status, and household income were added as covariates. In model 2, data on the patients’ initial diagnosis as assessed by a coronary specialist—pectoris angina, acute myocardial infarction, an ischemic heart disease, or another coronary disease—were added to model 1. In model 3, other potential CVD risk factors—BMI, physical activity, and awareness of tobacco as a cardiovascular risk factor—were added to model 2. In model 4, an interaction term composed of household income and awareness of tobacco as a CV risk factor was added to model 3. The introduction of the interaction term allowed us to assess the magnitude of the association between household income and smoking cessation by awareness of tobacco as a CV risk factor, as well as enabled us to study the effect modification of this magnitude. Here, we have mainly analysed the coefficients of the interaction term as a modification of the effect of income by awareness of tobacco as a CV risk factor.

All statistical analyses were performed using SAS 9.3 software (SAS Institute).

## Results

In total, 1289 patients participated in the follow-up study, of which 250 were ever smokers. The follow-up group of respondents who were smokers was not significantly different from the non-respondents who were smokers for all characteristics, except for physical activity (Table 1). Compared with the smokers in the initial study (*n* = 1001), the smokers in the follow-up study were more likely to have a Luxembourg nationality, to be married or living with a partner, and to have regular physical activity (Table 1). Of the 250 participants who were current smokers at the time of the initial study, 43.2% indicated that they still smoked, while 56.8% had stopped smoking by the follow-up (Table 2). Smoking cessation was not significantly associated with gender, age, nationality, marital status, or awareness of tobacco as a CV risk factor, but was significantly associated with income, initial diagnosis, and health-related behaviour (practicing regular physical activity and BMI) by the follow-up.

**Table 1:** Characteristics of smokers in 2008/2009, and comparison between respondent smokers and non-respondent smokers

	n	2008/2009 (*n* = 1001)%	Respondents 2013/2014 (*n* = 250)%	Non respondents (*n* = 751)%	*p*-value (*)
Sex					
Men	751	75.0	75.6	74.8	0.81
Women	250	25.0	24.4	25.2	
Age					
Less than 54	399	39.9	40.8	39.6	0.73
55–64	314	31.4	32.4	31.0	
65 and more	288	28.8	26.8	29.4	
Nationality					
Luxembourg	564	66.9	70.3	65.5	0.18
Other	279	33.1	29.7	34.5	
Marital status					
Married / partnership	508	63.7	70.9	61.2	0.0124
Other	290	36.3	29.1	38.9	
Diagnosis					
Pectoris Angina	381	38.1	38.0	38.1	0.88
Acute Myocardial Infarction	155	15.5	16.8	15.1	
Ischemic Heart Disease	131	13.1	12.0	13.5	
Other	334	33.4	33.2	33.4	
BMI					
Normal	317	32.5	31.6	32.7	0.51
Overweight	395	40.4	43.4	39.4	
Obese	265	27.1	25.0	27.8	
Physical Activity					
Regular physical activity	187	23.0	33.6	19.0	<0.0001
Occasional physical activity	216	26.5	27.3	26.3	
No physical activity due to health	148	18.2	12.7	20.2	
No physical activity	263	32.3	26.4	34.5	
Awareness of tobacco as a CV risk factor					
Yes	350	35.0	37.6	34.1	0.31
No	651	65.0	62.4	65.9	

(*) *p*-value concerns the comparison of respondent smokers’ characteristics with those of non-respondent smokers

**Table 2:** Change in smoking status between 2008/2009 and 2013/2014 (among those who reported smoking in 2008/2009)

	Tobacco			
	Ever smokers (N)	Current smokers (%)	Former smokers (%)	*p*-value
All	250	43.2	56.8	
Sex				
Men	189	43.4	56.6	0.92
Women	61	42.6	57.4	
Age				
Less than 54	53	41.5	58.5	0.96
55–64	92	43.5	56.5	
65 or older	105	43.8	56.2	
Nationality				
Luxembourg	175	44.0	56.0	0.76
Non-Luxembourg	75	41.9	58.1	
Marital status				
Married/Partnership	168	41.1	58.9	0.29
Other	81	48.2	51.8	
Household income (annual, in €)				
Less than 35,999	111	51.4	48.7	0.07
Between 36,000 and 53,999	75	37.3	62.7	
54,000 or more	64	36.5	63.5	
Diagnosis				
Pectoris angina	95	44.2	55.8	0.0062
Acute myocardial infarction	42	21.4	78.6	
Ischemic heart disease	30	40.0	60.0	
Other event	83	54.2	45.8	
BMI				
Normal	58	58.2	41.8	0.0093
Overweight	108	44.1	55.9	
Obese	84	31.7	68.4	
Physical activity				
Regular physical activity	100	43.8	56.3	0.0270
Occasional physical activity	58	26.8	73.2	
No physical activity due to health	49	46.8	53.2	
No physical activity	43	56.1	43.9	
Awareness of tobacco as a CV risk factor				
Yes	178	44.3	55.7	0.58
No	74	40.5	59.5	

*CVD* Cardiovascular disease, *BMI* Body mass index

In Table 3, awareness of tobacco as a CV risk factor is shown in relation to patient characteristics and smoking status. The awareness of tobacco as a CV risk factor was associated with age, household income, and BMI for all smokers. For former smokers, awareness of tobacco as a CV risk factor was significantly associated with age, household income, and physical activity. In current smokers, this awareness was only associated with nationality and household income.

**Table 3:** The distribution of tobacco as a CV risk factor depending on smoking status

	ALL		Current smokers		Former smokers	
	Awareness of tobacco as a CV risk factor	*p*-value	Awareness of tobacco as a CV risk factor	*p*-value	Awareness of tobacco as a CV risk factor	*p*-value
All	70.4		72.2		69.0	
Sex						
Men	71.4	0.53	73.2	0.69	70.1	0.63
Women	67.2		69.2		65.7	
Age						
Less than 54	88.7	0.0031	90.9	0.06	87.1	0.0460
55–64	68.5		72.5		65.4	
65 or older	62.9		63.0		62.7	
Nationality						
Luxembourg	69.7	0.76	66.2	0.0285	72.5	0.16
Non-Luxembourg	71.6		87.1		60.5	
Marital status						
Married/Partnership	71.4	0.57	69.6	0.41	72.7	0.12
Other	67.9		76.9		59.5	
Household income (annual, in €)						
Less than 35,999	64.0	0.0009	73.7	0.06	53.7	0.0009
Between 36,000 and 53,999	64.0		57.1		68.1	
54,000 or more	88.9		87.0		90.0	
Diagnosis						
Pectoris angina	69.5	0.73	71.4	Non applicable	67.9	0.33
Acute myocardial infarction	64.3		55.6		66.7	
Ischemic heart disease	73.3		100.0		55.6	
Other event	73.5		68.9		79.0	
BMI						
Normal	65.5	0.10	65.6	0.29	65.2	0.16
Overweight	66.7		73.3		61.4	
Obese	79.8		84.0		77.8	
Physical activity						
Regular physical activity	77.1	0.37	71.4	0.70	81.5	0.091
Occasional physical activity	73.2		86.7		68.3	
No physical activity due to health	63.8		72.7		56.0	
No physical activity	68.3		73.9		61.1	

*CVD* Cardiovascular disease, *BMI* Body mass index, *€* Euro

In Table 4, the full results from all three models without the interaction term are presented. In the first multivariate logistic model, quitting smoking was associated with income after controlling for socio-demographic indicators (sex, age-group, nationality, marital status). The odds of smoking cessation were higher among patients belonging to the two highest income groups (odds ratio [OR] = 2.0; 95% CI, 1.1–3.8 and OR = 2.0; 95% CI, 1.0–4.0, respectively) than among those belonging to the lowest income group.

**Table 4:** Logistic models for the probability of smoking cessation (*n* = 250)

	Model 1		Model 2		Model 3	
	OR	CI 95%	OR	CI 95%	OR	CI 95%
Sex						
Men	ref.		ref.		ref.	
Women	1.21	[0.66–2.22]	1.42	[0.76–2.68]	1.94	[0.88–4.27]
Age						
Less than 54	1.13	[0.57–2.27]	1.10	[0.54–2.25]	1.15	[0.51–2.61]
55–64	1.03	[0.58–1.85]	0.85	[0.46–1.57]	0.94	[0.47–1.85]
65 or older	ref.		ref.		ref.	
Nationality						
Luxembourg	ref.		ref.		ref.	
Non-Luxembourg	1.43	[0.78–2.62]	1.46	[0.78–2.74]	1.45	[0.73–2.90]
Marital status						
Married/Partnership	1.24	[0.71–2.15]	1.30	[0.73–2.32]	1.39	[0.72–2.67]
Other	ref.		ref.		ref.	
Household income (annual, in €)						
Less than 35,999	ref.		ref.		ref.	
Between 36,000 and 53,999	**2.01**	**[1.06–3.81]**	**2.05**	**[1.05–3.99]**	**2.85**	**[1.32–6.15]**
54,000 or more	**2.04**	**[1.03–4.02]**	**2.56**	**[1.25–5.24]**	**2.83**	**[1.24–6.46]**
Diagnosis						
Pectoris angina			1.56	[0.84–2.89]	1.66	[0.83–3.29]
Acute myocardial infarction			**5.54**	**[2.23–13.73]**	**5.35**	**[1.99–14.36]**
Ischemic heart disease			2.02	[0.81–5.02]	2.88	[0.96–8.63]
Other event			ref.		ref.	
BMI						
Normal					ref.	
Overweight					2.09	[0.96–4.54]
Obese					**3.69**	**[1.58–8.61]**
Physical activity						
Regular physical activity					1.60	[0.70–3.67]
Occasional physical activity					**2.90**	**[1.13–7.46]**
No physical activity due to health					1.51	[0.58–3.93]
No physical activity					ref.	
Awareness of tobacco as a CV risk factor						
Yes					0.54	[0.27–1.09]
No					ref.	

*CVD* Cardiovascular disease, *OR* Odds ratio, *CI* Confidence interval, *BMI* Body mass index, *€* Euro

The bold numbers indicate a significant association to the error threshold of 5%.

Adding diagnostic factors (acute myocardial infarction, ischaemic heart disease, and angina pectoris) in the second model did not alter the odds of smoking cessation among patients belonging to the two highest income groups (OR = 2.1; 95% CI, 1.1–4.0 and OR = 2.6; 95% CI, 1.3–5.3, respectively). In the third and full model, risk behaviour elements (BMI, physical activity) were added as cardiovascular risk factors. The odds of quitting smoking among patients belonging to the two highest income categories remained significantly higher compared with patients belonging to the lowest income group (OR = 2.8; 95% CI, 1.3–6.1 and OR = 2.8; 95% CI, 1.2–6.5). Awareness of tobacco as a CV risk factor was not associated with the probability of smoking cessation.

In Table 5, an interaction term was added to model 3. The odds of quitting smoking were only associated with income when patients were aware of tobacco as a CV risk factor. Indeed, among patients who were aware that tobacco is a CV risk factor, the odds of smoking cessation were 5.62 (95% CI: 2.13–14.86) and 3.65 (95% CI: 1.51–8.86) times in patients with household incomes of 36,000–53,999 euros and ≥54,000 euros, respectively, compared to patients with an household income of <36,000 euros.

**Table 5:** Logistic models for the probability of smoking cessation with interaction income and awareness of tobacco (adjusted for sex, age, nationality, marital status, diagnosis, BMI, and physical activity; *n* = 250)

	Model 3	
	OR	CI 95%
Household income (annual, in €)		
Less than 35,999	ref.	
Between 36,000 and 53,999	0.82	[0.23–2.85]
54,000 or more	1.46	[0.18–11.73]
Awareness of tobacco as a CV risk factor		
Yes	**0.25**	**[0.09–0.66]**
No	ref.	
Interaction Household Income x Awareness of tobacco		
Between 36,000 and 53,999 vs Less than 35,999 at Awareness of tobacco = yes	**5.62**	**[2.13–14.86]**
54,000 or more vs Less than 35,999 at Awareness of tobacco = yes	**3.65**	**[1.51–8.86]**

*CVD* Cardiovascular disease, *OR* Odds ratio, *CI* Confidence interval; *BMI* Body mass index, *€* Euro

The bold numbers indicate a significant association to the error threshold of 5%.

## Discussion

This study showed an independent association between smoking cessation and income status among adult patients with CVD 5 years after an angiography in Luxembourg. Patients belonging to the higher income groups were more likely to quit smoking compared to patients belonging to the lowest income group, and this association held even after accounting for diagnostic factors and cardiovascular risk factors or awareness of tobacco as a CV risk factor. These results are similar to those found in other studies examining educational level or occupational status as a proxy for socio-economic status [15, 18–20]. People with a low socio-economic status are thus confronted with simultaneous problems: they have higher rates of coronary heart diseases and a higher prevalence of smoking, as well as a higher risk of relapse and a decreased likelihood of successfully quitting smoking [15, 20]. It is also important to stress that the association between socio-economic status—regardless of the measure (i.e. income, educational level, or occupational status)—and smoking cessation in coronary patients is independent of other well-known predictors of successful cessation or relapse.

However, for this analysis, household income was selected as a proxy for socio-economic status instead of educational level because a majority (around 80%) of the participating patients (in both the initial and follow-up study) were over 55 years old making educational level less relevant as a socio-economic indicator when compared with income. Furthermore, although smoking initiation has been shown to be strongly related to educational level, smoking cessation has been shown to be strongly related to income [15, 21]. Indeed, the association found between smoking cessation and income status in patients with CVD in this study supports the findings in the general population. For example, studies examining socio-economic status and smoking cessation in Western countries generally indicate that smokers from lower income groups are less likely to be successful in quitting smoking [22]. Furthermore, in hospitals, income-related inequalities in smoking cessation may be due to the degree of patient adherence to advice or recommendations regarding health behaviour change. Indeed, patients with chronic conditions belonging to a low-income category were less likely to adhere to the recommendations to reduce or stop smoking [23], and our results suggest that having been admitted to a hospital for a coronary event is less effective in serving as a “wake-up call” for patients with a low income.

Although awareness of tobacco as a CV risk factor is necessary for a behavioural change, it is not a sufficient reason to quit smoking, particularly for individuals in lower income groups. In our study, awareness of tobacco as a CV risk factor was not significantly associated with the probability of smoking cessation, even when we only adjusted for age and sex. However, by assuming an interaction between the income level of patients and their awareness of the effects of smoking on CVD, we obtained different results, which allowed us to understand one of the mechanisms that may explain the association between smoking cessation and income in patients with CVD. Indeed, the awareness of tobacco as a CV risk factor appears to play a mediating role (or has a modifying effect) between income and smoking cessation. Income was only associated with smoking cessation among patients who were aware of the health risks of tobacco. In addition, smokers with higher or medium incomes who were aware of tobacco as a CV risk factor were more likely to quit smoking.

### Strengths

This database offers a unique opportunity to assess smoking cessation in a very particular group of patients: people with cardiovascular problems 5 years after a coronary angiography. It also allows the assessment of socio-economic inequalities in smoking cessation using a secondary prevention perspective. Studies have shown that socially disadvantaged patients are less likely to participate in rehabilitation care programs after a cardiovascular event [24]. However, income is rarely used as a socio-economic indicator in studies concerning smoking or smoking cessation in Europe [15]. Furthermore, smoking cessation in patients with CVD is the most effective secondary preventive measure in reducing mortality [25, 26]. Therefore, our study helps to fill a gap in the literature and provides guidance for future intervention programmes.

### Limitations

Only a minority (30%) of the patients participated in the follow-up survey. Therefore, for the majority of the original participants, current smoking status is unknown. Furthermore, information concerning the number of patients that died during the follow-up period is based on responses by relatives and not on the official vital status in the national register. Therefore, this number may be (substantially) higher and might partially explain the high number of non-responders.

A comparison of the characteristics of respondents who were smokers and non-respondents who were smokers only showed a difference in marital status and physical activity.

In addition, data regarding smoking status were only collected at the time patients were admitted to the hospital (2008/2009) and at the time of follow-up, 5 years later. Patients were not asked whether they had already tried to stop smoking during this period, so the rate of failed attempts to stop smoking is unknown. Also unknown is at what time ex-smokers stopped smoking. It might be that former smokers stopped smoking just after admission to the hospital making the 5-year follow-up period rather artificial with very few quitters in the last part of this period. However, such limitations are inherent to most follow-up studies because it is often difficult to obtain unbiased information on the changes in behaviour regarding smoking in between interval surveys [27].

Furthermore, most data used in this analysis were based on self-reports. Therefore, smoking cessation might have been overestimated since it is influenced by social desirability. However, measurement of smoking behaviour by self-report has been shown to correlate well with cotinine testing in most studies [28], and self-reported smoking cessation is a reliable method in assessing smoking status in patients admitted to hospitals [25, 29].

Finally, the information regarding income was quite crude. Only information on large household income groups was available, and for a substantial number of participating patients, information on income was unknown. The imputation procedure applied to deal with the absence of data on income was rather basic, and given the absence of data on household composition, it was not possible to calculate the equivalised household income, which would have facilitated comparing income. Moreover, given the fact that data on income were only available at follow-up, it was not possible to assess changes in income position during the follow-up period.

## Conclusions

Our study showed evidence of socio-economic inequalities in smoking cessation 5 years after a coronary angiography, where quitting smoking is an important measure of secondary prevention in patients with CVD. Indeed, quitting smoking reduces the risk of recurrent events, improves patient quality of life, and is cost-effective [30, 31]. This study also highlighted the influence of income on adopting positive behaviours regarding CVD risk factors (such as smoking) and showed that patients in the highest income groups were more likely to quit smoking. This influence may be explained by the existence of a significant interaction between awareness of tobacco as a CV risk factor and the level of income. Given the persistence of a relatively high level of smoking among people at high risk, significant efforts should be made to encourage and support smokers to quit smoking in order to improve social welfare and substantially reduce morbidity and premature death associated with tobacco. Thus, it is important that practitioners develop intervention strategies that enable disadvantaged patients to stop smoking. In light of the benefits of smoking cessation on health and society, intervention strategies combining behavioural and pharmacological therapies should be implemented in major health facilities and should target lower income groups.

## Abbreviations

BMI: Body mass index
CDV: Cardiovascular diseases
CI: Confidence interval
INCCI: National Institute for Cardiac Surgery and Interventional Cardiology
OR: Odds ratio
